# Nanoscale trace metal imprinting of biocalcification of planktic foraminifers by Toba’s super-eruption

**DOI:** 10.1038/s41598-020-67481-w

**Published:** 2020-07-03

**Authors:** L. Lemelle, A. Bartolini, A. Simionovici, R. Tucoulou, W. De Nolf, F. Bassinot, T. de Garidel-Thoron

**Affiliations:** 1 Univ Lyon, ENS de Lyon, Univ Claude Bernard, CNRS, LGL-TPE, 46 allée d’Italie, 69342 Lyon, France; 2 Muséum National D’Histoire Naturelle, Département Origines & Evolution, CR2P MNHN, CNRS, Sorbonne Université, 8 rue Buffon CP38, 75005 Paris, France; 3 ISTerre, Univ. Grenoble Alpes, CNRS, CS 40700, 38058 Grenoble Cedex 9, France; 4 ESRF-The European Synchrotron, ID21/ID16B beamlines, 71 avenue des Martyrs, CS40220, 38043 Grenoble Cedex 9, France; 5 Institut Pierre-Simon Laplace/Laboratoire Des Sciences du Climat Et de L’Environnement, UMR 8212, CEA-CNRS-UVSQ, 91190 Gif-sur-Yvette, France; 6 Aix-Marseille Univ, CNRS, IRD, Collège de France, INRAE, CEREGE, 13545 Aix-en-Provence cedex 4, France; 7 Institut Universitaire de France (IUF), Paris, France

**Keywords:** Palaeontology, Imaging techniques

## Abstract

Bioactive metal releases in ocean surface water, such as those by ash falls during volcanic super-eruptions, might have a potentially toxic impact on biocalcifier planktic microorganisms. Nano-XRF imaging with the cutting-edge synchrotron hard X-ray nano-analysis ID16B beamline (ESRF) revealed for the first time a specific Zn- and Mn-rich banding pattern in the test walls of *Globorotalia menardii* planktic foraminifers extracted from the Young Toba Tuff layer, and thus contemporaneous with Toba’s super-eruption, 74,000 years ago. The intra-test correlation of Zn and Mn patterns at the nanoscale with the layered calcareous microarchitecture, indicates that the incorporation of these metals is syngenetic to the wall growth. The preferential Mn and Zn sequestration within the incipient stages of chamber formation suggests a selective incorporation mechanism providing a resilience strategy to metal pollution in the test building of planktic foraminifers.

## Introduction

Planktic foraminifers are one of the major constituents of calcium carbonate accumulated in pelagic seafloor sediments (30–80% of total deep-marine calcite budget), playing a crucial role in the carbon cycle and climate regulation^1,2^. Their calcitic tests with intricate and beautiful morphologies are easily preserved in marine sedimentary records and constitute an invaluable archive of past environmental and climatic conditions. The trace metal contents of these tests are widely used as palaeoceanographic and palaeoclimatic proxies, since metal incorporation is strongly affected by the environmental conditions in which the organisms grew^3^. Differences in trace element contents between calcitic foraminifer tests and inorganic calcite precipitated in the same seawater conditions show that biological processes play an important role in metal incorporation^4^. A detailed examination of trace metal distribution within the shell walls may help in identifying the biological processes controlling their incorporation. The most studied and emblematic example is the Mg/Ca paleothermometer proxy, whose intra-test micro-distribution displays a typical banding pattern in calcareous perforate foraminifers, which include planktic and benthic species. Different biological processes have been proposed to explain it^5–8^. It has been shown that Mg incorporation for some planktic foraminifer species is diurnally paced and modulated by light–dark cyclicity^7,8^ and is assumed to be related to physiological processes, such as symbiont photosynthesis or respiration, that affect the carbonate chemistry at the site of calcification^5^, or to a mitochondrial uptake of Mg^7^. In addition to Mg, banding patterns of other minor and trace elements (Na, Sr, K, S, P, N, B, Mn) have also been displayed from culture experiments or found in live calcareous perforate foraminifers retrieved from natural seawater environments^9–13^.

This study aims to unravel the intra-test multi-intake of trace metals, such as Mn and Zn, into planktic foraminifera tests. As Mn and Zn contents in foraminifers reflect the availability of free Mn^2+^ and Zn^2+^ in seawater^3,14–20^, this study focuses on tests of organisms grown and adapted to surface seawaters with high Mn and Zn in the context of major volcanic eruptions.

During volcanic eruptive events, planktic foraminifers can be exposed to releases of these bioactive metal cations by leaching of acid aerosols and metal salts adsorbed on the surface of ash, when ash-loaded falls come into contact with ocean surface waters^21,22^. Mn^2+^ and Zn^2+^, essential to biological processes, can stimulate the primary productivity and enzymatic activity of phytoplankton^21^. If in excess, such as in the case of a super-eruption, a toxic impact on sensitive marine organisms, such as biocalcifying microorganisms, has been suggested^23^. On the other hand, culture experiments have shown that foraminifers can grow and produce new chambers, even in culture medium enriched in metals such as Mn, Cu, and Zn^16,24,25^. The calcitic layers produced during growth record these metal enrichments. It has therefore been proposed that metal content in foraminifer tests might be useful monitoring tools for both anthropogenic and volcanic metal marine pollution^16,24,25^. Yet the mechanism of incorporation of metals such as Zn and Mn during the formation of a new planktic foraminifer chamber has to be explored, especially in response to large volcanic eruptions. For this purpose, we investigate tests picked from the Young Toba Tuff layer, which are contemporaneous to the greatest volcanic cataclysm of the Quaternary: the Toba super-eruption, 74,000 years ago^26^. We extracted exceptionally well-preserved fossil foraminifer tests from the Young Toba Tuff layer, in marine sediment core BAR94-25 (water depth 1558 m bsl). This core was collected in the Andaman Sea (Eastern Indian Ocean) relatively close (~ 600 km North-West) to the Toba Caldera Complex, located North of Sumatra^27^.

We focus on the *Globorotalia menardii* (Parker, Jones & Brady, 1865) species, which is relatively abundant in tropical to subtropical Indian Ocean waters and whose shell is dissolution resistant^2^. Given the huge quantities of ashes ejected by Toba’s super-eruption (i.e. ~ 2,800 km^3^ of magma)^26^, high fluxes of ash leachate metal elements might have contaminated the ocean surface waters, particularly in the proximal area of the volcano.

Our working hypothesis posits that intakes of high levels of bioactive metals from ocean waters fouled by Toba’s super-eruption may have triggered specific incorporation patterns into foraminifers’ tests, which can be detected and mapped through nano-imaging. Such patterns might fingerprint some of the processes implicated in Mn and Zn incorporation, and, at the same time, might unravel perturbations and/or resilience strategies in test build-up in response to Toba’s metal pollution of the ocean. In well-preserved planktic foraminifer tests, the growth stages are recorded in the layered ultrastructure of chamber walls^28^. Thus, we explore the trace metal distribution at the nanoscale over the entire width of the calcitic wall sub-micrometer structures of the last chamber of *G. menardii* tests. We aim to describe at the nanoscopic scale, the trace metal incorporation occurring during the test chamber formation and its interplay with the build-up sequence.

To unravel the Mn and Zn trace patterns, a nanometric spatial resolution and high elemental sensitivity by nano-imaging is required, and is achieved here by synchrotron X-ray Fluorescence analyses (XRF)^29^. In previous works, foraminifer intra-test elemental distribution mapping reached spatial resolutions of 85–500 nm for elemental concentrations at the 100 ppm level (nanoscale Secondary Ion Mass Spectrometry—NanoSIMS), or of 1 µm with minor element sensitivities (Synchrotron micro-XRF and Electron Probe Micro Analysis—EPMA) for concentrations relative to the average Ca signal^12,13^. We have focused our nano-XRF measurements on two sampling choices of the last chamber walls: full wall bulk (10 to 11 µm thick) and patterns in transverse sections across the wall (~ 1 µm thick). Unraveling the Mn and Zn trace patterns also required optimized sample preparations, including FIB preparations for synchrotron X-ray Fluorescence (XRF) nano-imaging^29^. Thin cross-sections of homogeneous thickness (1 ± 0.1) were cut perpendicularly to the test chamber’s surface (following the straight pore channels) so the beam could traverse them for high lateral resolution (up to 50 nm) XRF trace element mapping with negligible depth effects. These analyses were performed on the hard X-ray ID16B nanoprobe^30^ of the European Synchrotron Radiation Facility (ESRF). A combination with imaging on the tender X-ray microprobe ID21 (ESRF) was carried out to compare these trace metal patterns to those of the more abundant constitutive low atomic number (Z) elements of the wall (Mg, P, S, Ca).

## Results

### Preliminary wall bulk XRF-imaging

In total, six specimens of *Globorotalia menardii* were successfully mapped: five fossils collected from the Young Toba Tuff layer (T_YTT1_-T_YTT5_) and a live specimen which thrived in subsurface waters south west of Sumatra (T_IND_) (see “Materials and methods” for more details).

**Figure 1:**
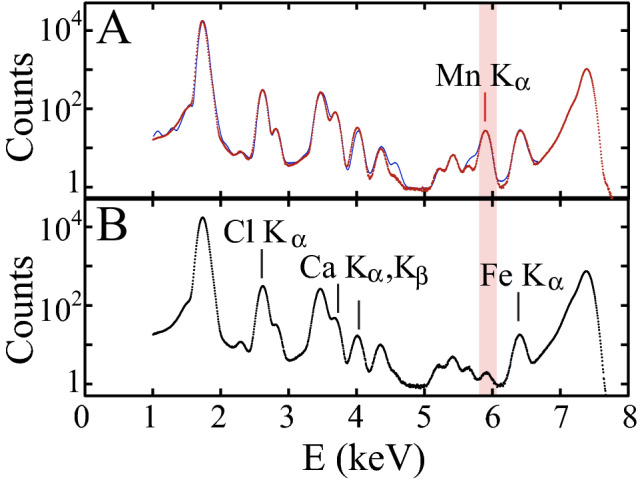
Mn from the last chamber of *Globorotalia menardii.* (**A**) Average XRF spectrum compiled from a µ-XRF map recorded at 7.3 keV (dwell-time is 5 s, scan step size is 250 nm) on a FIB cut of the last chamber wall of a *Globorotalia menardii* extracted from the YTT level of the specimen T_YTT1_ (BAR94-25 core, 307 cm depth). (**B**) Same measurement on a test of *Globorotalia menardii* sampled live in the Indian Ocean water column of the specimen T_IND_ (Gyrafor-B, St.C, T3N4-2F). The main K_α_ lines of the samples are reported and the Mn K_α_ line is in the red zone. The Mn K_α_ raw counts were compiled using PyMCA indicating Mn contents in YTT specimen wall chamber 5 to 10 times higher than in the living water column specimen wall chamber. Note that Fe K_α_ shows close counts in A and B.

**Figure 2:**
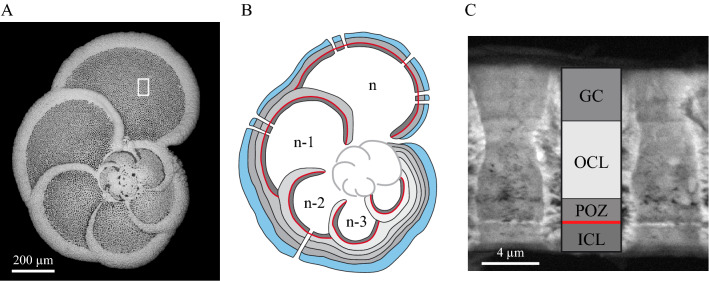
Wall structure of the last chamber of *Globorotalia menardii*. (**A**) Secondary Electron Scanning Electron Micrograph (SE-SEM) of the *G. menardii* T_YTT5_ specimen coming from the YTT level (BAR94-25 core, 318 cm depth). The white rectangle corresponds to the zone where the fragment T_YTT5_ (Fig. 2C) was collected. (**B**) Classical diagram of planktic foraminifer test construction via the sequential addition chambers: with the formation of every newly secreted chamber, the whole of the pre-existing test is covered by a new OCL^28^. The black lines represent the organic linings, the red line the POS zone (Primary Organic Sheet, starting point of the shell construction), the dark-gray layer the Inner Calcitic Layer (ICL), the light-gray layers the Outer Calcitic Layers (OCLs), the light-blue layer the Gametogenic Crust (GC). (**C**) SE-SEM image of the fragment T_YTT5_ coming from the last chamber (**n**) of the *G. menardii* specimen illustrated in Fig. 2A and showing the ICL-POZ-OCL-GC growing structure of the wall described elsewhere in the text. The red line indicates the Primary Organic Sheet (POS) sensu stricto. The Primary Organic Zone (POZ) corresponds to the Ca-poor precursor layer (Fig. 3A) and it is adjacent to the thin POS (see text for more details). The GC has a distinct crystal structure with large elongated euhedral crystals, while the ICL and OCL consist of much smaller submicron crystallites^28^.

An average XRF spectrum compiled from µ-XRF maps recorded at 7.3 keV (T_YTT1_ FIB cut) displayed an enrichment in Mn with respect to that living specimen (T_IND_) retrieved from the water column in the Indian Ocean (Fig. 1). An average XRF spectrum of the nano-XRF maps was recorded at 17.4 keV across wall fragments of the last chamber from 3 specimens collected from the Young Toba Tuff layer (T_YTT2_, T_YTT3_ and T_YTT4_) (Fig. S1A). They yielded an average Mn/Ca ratio value of 7.66·10^–4^ ± 3.09·10^–4^ (SD) wt%, and of 15.85 ± 6.33 (SD) wt% for Mn/Zn (see “Materials and methods”). Others metals of volcanic interest, such as Cu, Ni, Co, were difficult to estimate due to interferences from Ca lines pile-up. These nano-scale elemental mappings, performed on the test’s surface, avoid the diagenetic surface deposits^31,32^ (Fig. S1) but integrate the trace metal intra-test patterns over the whole wall depth of the fragments (“bulk” analyses). Then, the distribution of the major and detected trace elements was investigated across a perpendicular cross section of the test’s wall, to probe the multiple calcitic layers parallel to the surface (Fig. 2, Appendix 1).

### Wall ultrastructure revealed by the Ca and Sr distributions

**Figure 3:**
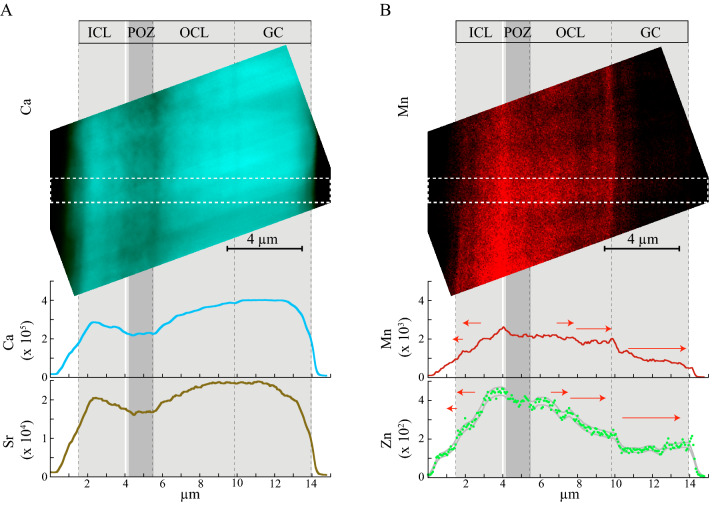
Mn-Zn distributions imprinted on the calcitic structure of the wall as seen by nano-XRF. (**A**) High spatial resolution map of the Ca K_α_ lines recorded at 17.4 keV on ID 16B ESRF (dwell-time is 3 s, pixel size is 60 nm) and the Ca and Sr profiles measured on the *G. menardii* last chamber T_YTT5_ fragment parallel to the axis of the ICL-POZ-OCL-GC wall growth structure (see corresponding SE-SEM image in Fig. 2C). Profiles have been averaged along a 21 pixel-wide rectangle (displayed by a white dashed line box). Data is reported in counts of fluorescence. The ICL-POZ-OCL-GC wall growth structure is indicated as gray boxes and displays a Ca-poor POZ, a Ca-rich GC and nano-bands in the ICL and OCL. Error bars are in the line widths, or comprised between gray lines. (**B**) Same representation for the Mn and Zn K_α_ lines displaying the map of Mn, and the Mn and Zn profiles highlighting the Mn-Zn rich POZ, the Mn-Zn poor GC and nano-bands in the ICL and OCL. Error bars are in the line widths. The red arrows indicate the bipolar growth direction of ICL, OCL and GC.

The nano-XRF imaging performed at 17.4 keV on the ID16B beamline of the ESRF (see “Materials and methods”) revealed distributions of the Ca major element and of the associated Sr alkaline-earth element in cross sections of a fragment of the last chamber of *G. menardii* test from the lowermost Young Toba Tuff layer (T_YTT5_ sample). The Ca K_α_ line XRF map displays a “three”-lamellar wall structure, composed of two Ca-rich layers encasing a Ca-poor layer 1–1.5 µm thick (Fig. 3A). The SEM image of the same T_YTT5_ cross section (Fig. 2C) shows that this Ca-poor layer is adjacent to the thin primary organic sheet (POS) defined sensu stricto as a thin (~ 100–130 nm) organic lamina^33^. It is bound upwards by a layer displaying a darker mesh corresponding to a lower atomic number (Z) contrast and/or a high porosity (Figs. 2C and 3A). The association of this Ca-poor layer with the POS lamina, is, by extension, referred to hereafter as the primary organic zone (POZ). The thickness and “frothy” fabric of POZ, as observed in the nano-XRF Ca map (Fig. 3), is consistent with the complex branched organic network previously observed in a ~ 700 nm thick intra-test layer of another planktic foraminifer species, *Orbulina universa* d’Orbigny 1839^11^. Thus, the Ca-poor POZ layer is here assumed to correspond to the precursor layer of chamber wall construction of *G. menardii*^28^ (see Appendix 1). In the nano-XRF Ca map, the Ca-rich layers coincide with the inner calcitic layer (ICL) and outer calcitic layer (OCL), clearly visible in the SEM images (T_YTT5_ fragment in Fig. 2C). They correspond to the subsequent stage of bi-directional wall growth (bilayer structure) on either side of the precursor layer (POZ) ^28^ (Fig. 2B, Appendix 1). Both the ICL and the OCL show small and sub-parallel intensity variations displaying several alternating large Ca-rich bands and thin Ca-poor bands a few hundred nm thick (Fig. 3A, Fig. S2). Frothy fabric is also observed in the Ca-poor bands on the internal side of both the ICL and the OCL (Fig. 3A). The OCL appears thicker than the ICL (Fig. 3A). The homogeneity of the outermost Ca-rich layer (lacking in thin Ca-poor bands) differentiates the OCL from the gametogenic crust (GC) deposited once the last chamber is formed, upon all the chambers (Fig. 2B and Appendix 1). This sharp boundary is also clearly noticeable in the T_YTT5_ SE-SEM photo (Fig. 2C). In summary, the nanoscale distributions of the Ca major element and of the associated Sr alkaline-earth element, configure the intra-test layered “ICL-POZ-OCL-GC” microarchitecture of the wall across the last chamber of *G. menardii* (Figs. 2C and 3A), inherited from the test’s morphogenesis (see Appendix 1).

**Figure 4:**
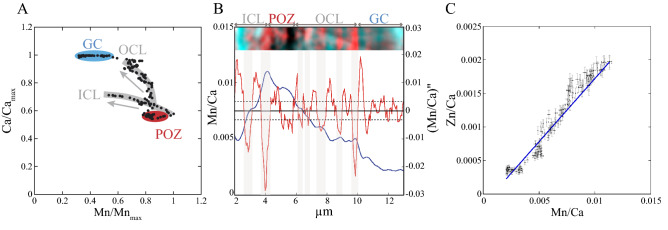
Zn, Mn and Ca correlations in the wall. Same data as in Fig. 3. (**A**) Relative variation of the Ca K_α_ plotted versus Mn K_α_ line fluorescence data showing from right to left the Ca-poor and Mn-rich POZ (in red), the continuous depletion trajectories in Mn of ICL and OCL parallel to the direction of growth (in gray) and the Ca-richest and Mn-poorest GC (in blue). The gray arrows indicate the growth direction of ICL and OCL. (**B**) The ratio of the K_α_ line fluorescence counts of Mn over Ca and its second derivative along the profile of Fig. 3. The relative Mn/Ca error bar of ca. 2% is in the line width. The values at ± $\sigma_{{\left( {S_{X} /S_{Y} } \right)^{\prime \prime } }}$ from zero (Eq. 4 in “Materials and methods”) are reported (horizontal dashed lines). Bands are detected events if contiguous fluctuations fall, away from these threshold values. The alternated white and gray bars between two zero values of the second derivative of the Mn/Ca ratio display a banded pattern, whose bands contain alternating positive and negative extrema, and thus minimal and maximal Mn/Ca ratio values. The color-coded image displays the Mn nano-banding (in red) in opposite phase to the Ca nano-banding (in turquoise) (Fig. S2). (**C**) Zn K_α_ plotted versus Mn K_α_ line fluorescence data, both normalized to the Ca K_α_ line fluorescence data showing a strong positive correlation and its linear fit (the Pearson correlation coefficient ρ = 0.975 and Zn/Ca ≈ 0.19 Mn/Ca).

Given the symmetry of the wall ultrastructure, the nano-XRF Ca map can be schematically summed-up through a compositional profile perpendicular to the surface of the wall (Fig. 3A) (“Material and methods”). The Ca profile reveals heterogeneity in the Ca distribution at the scale of the wall structure with a Ca-depleted POZ and the GC Ca-rich layer containing approximately 50% more Ca than the POZ (Fig. 4A). On both sides of the POZ, the Ca content increases by up to 20% within the encasing ICL and by up to 50% within the OCL; thus, the OCL is more enriched than the ICL (Fig. 4A). The intra-test heterogeneity was then accounted for by elemental profiles compiled along the same axis (Fig. 3). The Sr profile mimics the Ca profile (Fig. 3A). However, the Sr/Ca ratio is slightly lower (about 30%) in the OCL and GC layers than in the ICL and the POZ (Fig. S3).

### The Mn–Zn distribution patterns of the wall structures

The Mn–Zn profiles display highest Mn and Zn contents in the POZ anti-correlated with the Ca and Sr profiles (Fig. 3B). The Mn content decreases to 40% within the encasing ICL and to 20% within the OCL (Fig. 4A), while the lowest contents are observed in the GC layer (Figs. 3B and 4B). The Mn and Zn contents measured in each structure of a cross section are strongly correlated, with a Mn/Ca ratio more than 5 times higher than the Zn/Ca one (Fig. 4C). Both the ICL and OCL show small and sub-parallel intensity increases forming few hundred nm thin bands superimposed on the previously described main signal (Figs. 4B and S2). A close view of these small variations (Figs. 4B and S2) displays alternate thin Mn-rich and Mn-poor bands, of variable intensity, over ~ 1 µm thick, in opposite phases to those of the Ca-banding pattern. Similar nano-banding also characterizes the Zn distribution across the ICL and OCL (see the strong positive correlation between Zn and Mn in Fig. 4C).

**Figure 5:**
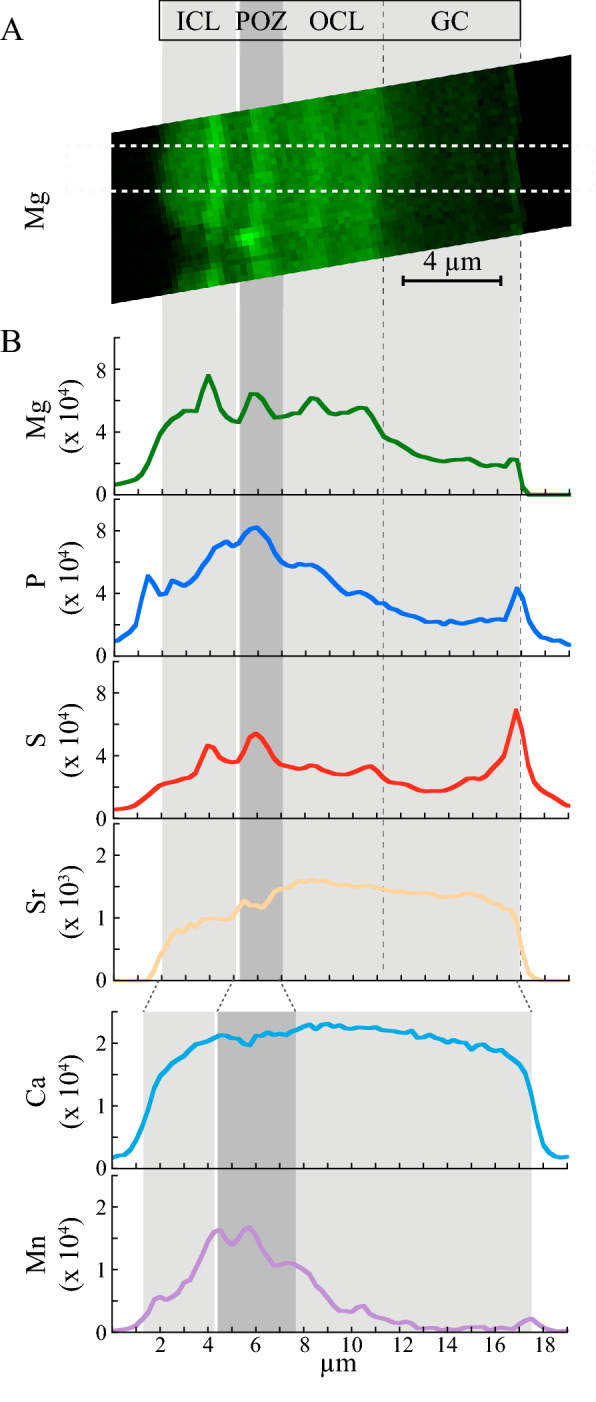
Mg-S-P-Mn distributions imprinted on the calcitic structure of the wall seen by micro-XRF. (**A**) High spatial resolution maps of the Mg K_α_ lines recorded at 2.55 keV on ID 21 ESRF (dwell-time is 10 s, step size is 250 nm, beamsize 300 × 300 nm^2^) and (**B**) the corresponding Mg, S, P and Sr profiles measured on a FIB section, cut perpendicularly to the surface of the last chamber of a *G. menardii* from the YTT level, T_YTT1_ (BAR94-25 core, 307 cm depth), see SE-SEM image in Fig. S5B. Spectra were averaged along a 10 pixel-wide rectangle. Data is reported in counts of fluorescence. The Ca and Mn profiles measured at 7.3 keV on the same sample and set-up were averaged along a 7 pixel-wide rectangle. The ICL-POZ-OCL-GC structure is indicated as gray boxes and displays a Ca-poor POZ, corresponding to the zone well visible in the T_YTT1_ FIB lamella SE-SEM image (Fig. S5B). Structures are enlarged and blurred at 7.3 keV due to the larger beamsize (1.2 µm). Error bars are in the line widths.

Micro-XRF imaging (ID21 beamline, ESRF) was performed at 7.3 keV to enhance the Mn K_α_ line XRF (ID21 beamline, ESRF). The Mn enrichment in and near the POZ, observed in the specimen T_YTT5_, was also confirmed at lower resolution for another specimen (T_YTT1_) picked from the Young Toba Tuff and for a modern specimen (T_IND_) on transverse, focused ion beam (FIB) cut across the last chamber (n) (Figs. 5 and S4). The localization of the POZ in the T_YTT1_ and T_IND_ was possible using the SEM images of the same FIB cuts on which µ-XRF maps were performed (Fig. S5). The POZ is much more prominent in the T_YTT1_ FIB cut than in the T_IND_ FIB cut (Fig. S5). It is noticeable that in the POZ of the section of the modern specimen (Fig. S4) the Mn enrichment is strikingly dampened compared to that of the fossil specimen from the YTT level (Fig. 5). The few hundred of nanometer-thick bands were not resolved as the Mn signal is approximately one order of magnitude lower than Toba's one (Fig. 1), so it could only be resolved in maps by statistical binning with a micrometric, rather than a nanometric, resolution.

The Mn distribution observed across the FIB cut perpendicular to the surface of the penultimate chamber (n-1) (Fig. S6) was also observed to be similar to that of the last chamber of the same test T_YTT1_ (Fig. 5). In first order, the main Mn enrichment is within and near the POZ, and, to a lesser extent, around the organic lining between the OCL (n-1) and OCL (n). The latter is supposed to be deposited during the last chamber (n) formation, according to the classical model of test construction via the sequential addition of chambers ^28^ (Fig. 2B).

### The Mg–S–P distribution patterns across the wall structures

We also investigated the Mg–S–P distribution across the ICL-POZ-OCL-GC wall structure (Fig. 5), as Mg^2+^, SO_4_^–2^, PO_4_^–2^ are known to play a role in the kinetics of calcite crystallization^34^. The distributions were recorded at 2.5 keV on the same FIB cuts (T_YTT1_ and T_IND_) as those on which the Mn distributions were investigated (Figs. 5, S4, S5) by micro-XRF imaging in vacuum at the ID21 beamline, to enhance the Mg, P and S XRF. The Mg K_α_ line fluorescence map (excited at 2.5 keV) and the corresponding profiles of the T_YTT1_ specimen exhibit a regular (same amplitude) periodic banding pattern (thin, high, and large, low Mg content ~ 2 µm thick bands). The S and Mg K_α_ profiles are strongly correlated (Fig. 5). The P K_α_ profile shows an intra-test enrichment with the richest values found within the POZ, in part similar to the Mn 7.3 keV profile (Fig. 5). The correlation between S and Mg with P is less obvious. We note, however, that the highest concentration of S matches the POZ, as observed for P.

In the FIB section of the modern specimen, enrichments in Mg and P of the POZ, and their decreasing contents in the ICL and OCL were also observed, but not the intra-CL Mg banding (Fig. S4). This is likely linked to a much thinner wall of the last chamber of the modern specimen (~ 5 µm) than that of the fossil YTT specimen (~ 15 µm) (Fig. S5). Moreover, modern specimens collected by nets in subsurface seawater most often do not present any gametogenic crust, implying that they have not fully completed their ontogenic development.

In conclusion, the POZ shows high enrichment in Mn, Mg and S*–*P in both modern and fossil YTT specimens (Figs. 3B, 5 and S4). In the thicker walls of the fossil YTT specimens, resolved differences of the Mg and Mn (Zn) banding patterns are displayed. While Mg enrichments comparable to that in the POZ are observed outside the POZ in the thin and high Mg bands of the ICL and OCL (Fig. 5), the Mn (Zn) pattern reveals a decreasing trend from the most enriched POZ toward the outer surfaces of the wall chamber.

## Discussion

The XRF imaging across the wall structure of the last chamber of both living and YTT fossil specimens of *G. menardii* revealed layered distributions of major (Ca), minor (Mg, Sr) and trace elements (Mn, Zn, S, P), following the growth ultrastructure (ICL-POZ-OCL). Besides inferring their preservation after post-depositional contamination or diagenesis, their comparison suggests implications regarding metal trace incorporation during wall build-up of a novel chamber and the final deposition of the GC, which are discussed below.

### The Mn and Zn versus Mg and Sr intra-test distribution

Several arguments support a minimal diagenesis of the fossil specimens extracted from the YTT. Although diagenetic influence was shown to account for some high values of bulk Mn/Ca ratios^31^, the Mn and Zn banding patterns observed here very likely result from a well preserved biological primary imprinting of their incorporation during the test building. First, we observed exceptional preservation of the YTT tests, likely due to rapid burial by very fine ashes. Moreover, the highest Zn and Mn enrichment of the intra-test POZ and the lowest one in the exterior GC (Figs. 3B, 4A and 5) argue against a strong diagenetic imprinting. The typical Mn diagenetic enrichments for core top specimens appear on the outer^35^ or on the inner test surfaces^32^. Lastly, high contents of Mn and Zn in the POZ are associated with high contents of Mg and Sr (Figs. 5 and S3). Mg- and Sr-rich phases in foraminifer tests have been shown to be vulnerable in case of post-depositional diagenetic processes such as dissolution and overgrowth^35,36^. The formation of Mn enrichments by an early diagenetic calcite dissolution associated with Mn rich recrystallization overgrowths would thus also imply a depletion in Sr and Mg that is not observed.

### Comparing Mn (Zn) and Mg patterns

The parallel Mn–Zn bands (Figs. 3B and S2) and Mg ones (Fig. 5A,B) were observed within the inner calcitic layer (“ICL”) and outer calcitic layer (“OCL”) and are referred to here as intra-calcitic layer (“intra-CL”) banding. It indicates that Mn, Zn and Mg incorporations have likely been carried out during the “bilayer” wall-thickening of a new chamber.

In *G. menardii* the intra-CL Mg banding is clearly distinct from the inter-calcitic layer (inter-CL) Mg banding, the latter being associated with the subsequent formation of other chambers (Figs. 2B and S6). In the case of inter-CL Mg banding, the high and thin Mg bands are observed within or close to the “outer organic linings”, intercalated with the outer calcitic layers (OCLs)^6,12,13,37^. Previously, the “intra-CL” Mg banding was considered as an exception for the spherically-shaped planktic foraminifer *O. universa*, whose last chamber was known to continue thickening after its formation^5,7^. Later, it was also documented for the last chamber of the trochospirally-shaped planktic foraminifer *Neogloboquadrina dutertrei* (d’Orbigny, 1839)^8^. Here we show that it is also present in the last chamber of planktic foraminifer *G. menardii*. Thus, the “intra-CL” Mg banding would seem to be a general feature of planktic foraminifera calcification, not yet revealed in benthic foraminifera.

The “intra-CL” Mg banding can be related to wall-thickening rate discontinuities and/or to different CaCO_3_ precipitation phases during the wall build-up of a new chamber^38^ (see the “intra-CL” Ca-bands in Fig. 3A). Culture experiments have ascertained that the “intra-CL” Mg banding in *O. universa* and *Neogloboquadrina dutertrei* is controlled by the circadian activity, whereby a wide, low Mg/Ca-calcite band forms during the day and a thin high Mg/Ca- calcite band forms at night^7,8^. Despite some species-specific differences, assuming a similar daily paced process for the intra-CL Mg banding for *G. menardii* (Fig. 5) would imply that once a new chamber is formed, its wall can continue to thicken over a few days (2–3 days in the case of the specimen in Fig. 5).

Compared with the Mg banding, the Mn (Zn) pattern shows substantial differences. The Mg banding has the same amplitude variations within the “intra-CL” layer (Fig. 5), while the Mn (Zn) pattern reveals a marked decreasing amplitude trend from the inner to the outer sides of the wall chamber (Figs. 4B and 5). Superimposed on this trend, an “intra-CL” nano-Mn (Zn) banding was displayed and its periodicity is indicated by the second derivative of Mn/Ca (Zn/Ca) along the profile (Fig. 4B). As for Mg, such periodic Mn (Zn) banding might also be related to a wall-thickening rates discontinuity, namely breaks and reactivations of the growth (see the “intra-CL” Ca-bands in Figs. 3A and S2). However, compared with the reported day and night cyclicity of the Mg bands detected at the micrometer scale, the cyclicity of the Mn (Zn) bands were detected at the nanometer scale (Figs. 3B, 4B and S2). This is likely suggesting a cyclic Mn and Zn incorporation implemented over periods shorter than the circadian period. Elucidating the underlying biological processes of these bandings, that are still highly debated for the most investigated “intra-CL” Mg banding produced under controlled culture experiments^7,8^, is beyond the scope of this nano-XRF imaging study mainly based on fossil specimens. The comparison of the intra-CL Mg and Mn banding at the same nanometer scale would certainly bring new insights into the involved driving forces. Overall, the main component of the Mn and Zn pattern consists of a decreasing amplitude trend from the inner to the outer sides of the wall chamber and may originate from different processes with respect to the Mg banding, characterized by the same amplitude variations.

### A selective incorporation of Mn and Zn

A selective and incipient incorporation of the Mn and Zn in the POZ during the build-up of a new chamber is supported by several aspects of the intra-test Mn and Zn distributions. First, in both modern and fossil specimens, the highest Mn contents are localized in the POZ, which is inherited from the precursor layer (Figs. 3, 4, 5 and S4). Secondly, the highest Mn content is confined exclusively within and around the POZ. The amounts of Mn and Zn in the outer “inter-layer” Mn banding observed in the penultimate chamber related to the chamber addition process, are indeed negligible (Fig. S6).

Furthermore, the Ca and Mn (Zn) nano-XRF imaging revealed specific nano-fabrics of the different layers (Figs. 3 and S2). The incipient POZ shows a clear highest enrichment in Mn (Zn) and a frothy fabric, as evidenced by the Ca map (Figs. 3 and S2). The ICL and OCL, further formed by incremental growth on either side of the POZ, display an increasing homogeneity and decreasing Mn and Zn contents from the internal to the external side (Figs. 3 and 4). Finally, the final forming GC is composed of large and homogeneous euhedral calcite crystals^28^ and is also the most depleted layer in both Mn and Zn (Figs. 3B, 4 and 5). This suggests a co-evolution between the fabric (e.g. grain size) of the calcium carbonate precipitated during the wall growth and its Mn–Zn content, as already proposed in previous works on planktic foraminifer tests^39^.

In both modern and fossil specimens, the observed co-variation of P with Mn (Figs. 5 and S4) might be one of the keys to explaining the selective incorporation mechanism of the Mn and its interconnection with the crystallinity of the calcium carbonate. P is a highly reactive element generally present as phosphate in nature. The opposing roles of both organic and inorganic phosphates as crystallization inhibitors/initiators of biomineralization are well established^40^. The high presence of phosphate in the POZ template (Figs. 5 and S4) might activate the incipient CaCO_3_ deposition and at the same time stabilize it in a transient amorphous-metastable phase^41,42^. Recently, a metastable vaterite phase was detected in planktic foraminifer tests^43^. This highlights the presence of non-classical crystallization pathways involving amorphous-metastable phases that ultimately transform to calcite. It can then be hypothesized that the high P (phosphate) content shown in the POZ might be associated with the deposition of amorphous-metastable calcium carbonate^41,42^. Its disordered structure could accommodate higher levels of Mn and Zn than the crystalline polymorphs^44,45^, explaining their selective incorporation at the incipient phase of biocalcification.

### Implications of the selective incorporation patterns

It is well known that volcanic ash fallen into surface seawater is followed by rapid release of metal salts adsorbed on glass shards, dumping free bioavailable Mn^2+^ and Zn^2+^ cations along with other metal cations^22^. A large quantity of such metal cations was likely released into surface seawater during the exceptional YTT super-eruptions, when the fallen ash was almost at the scale of the entire Indian Ocean^26^.

The Mn XRF intra-test mapping of both modern and YTT fossil *G. menardii* specimens revealed a preferential incorporation of Mn in the POZ, the incipient stage of the build-up of the wall of a new chamber. A remarkable correspondence of a prominent POZ and high metal trace contents is observable in the YTT specimens (Figs. 5 and S5). In the living specimen a less marked POZ is observed and corresponds to lower metal trace contents (Figs. S4 and S5). In YTT specimens, the entrapment of Mn–Zn in excess in the first stage of chamber formation enables continuing with the growth of the bilayer wall, almost spared of the consequences of the seawater metal pollution. Regardless of whether the seawater metal contents are high (as for a super-eruption) or not, the involved sequence of the wall build-up ensures the nominal shape and ends systematically in *G. menardii,* by the growth of a external layer formed of elongated euhedral crystals of nearly pure calcite.

It has already been shown by culture experiments and in situ observations in anthropogenic polluted areas, that foraminifers can survive and biocalcify in seawater with high metal contents, including Zn and Mn contents^16,25^. In individuals cultivated in seawater with high metal contents (Zn, Cu, Pb, Hg), important cellular ultra-structural alterations have been observed, such as mitochondrial degeneration, but also thickening of inner-organic lining or cell membranes, as well as proliferation of abnormally large lipid droplets, these last ones hypothesized to sequester toxicants in order to protect cells^46^. The preferential sequestering of metals such as Mn and Zn in the POZ might explain the exceptional resilience of foraminifers to biocalcify, even in the case of large environmental metal pollutions, likely caused by the YTT super-eruption and also contribute to cell detoxification. Besides, this intra-test sequestration in between calcitic layers with low levels of impurities (less prone to dissolution) has important consequences on the preservation of the chemical volcanic fingerprint in planktic foraminifer test. This brings forth the planktic foraminifer tests as high-potential bio-archives of past ocean metal volcanic pollution and Mn-Zn paleoceanographic proxies.

## Materials and methods

*Samples.* Fossil *G. menardii* tests were handpicked from the > 315 µm sieved fraction of the Young Toba Tuff (YTT) level of the BAR94-25 core^27^, and examined via scanning electron microscopy to select the best-preserved ones and avoid those with signs of test dissolution, which could be affected by preferential leaching of trace elements. YTT samples investigated in this study were extracted from five tests, and they have been denoted in the text as T_YTT1_ (= BAR94-25, 307 cm depth), T_YTT2_ (= BAR94-25, 317 cm depth), T_YTT3_ (= BAR94-25, 315 cm depth), T_YTT4_ (= BAR94-25, 318 cm depth) and T_YTT5_ (= BAR94-25, 318 cm depth).

Live *G. menardii* were collected in subsurface waters (62 to 30 m) of the subtropical Indian Ocean during the 2007 Gyrafor B Cruise, SW of Sumatra at Station C (9° 30′S 92°25′E) ^47^. The CEREGE MultiNet Midi (HydroBios, Kiel, Germany), equipped with an opening–closing 100-μm mesh net was used. The shells were then dried at room temperature by placement on absorbent cardboard before being transferred to micropaleontology slides. Samples investigated in this study were extracted from one test, labeled T_IND_.

The selected fossils and living *G. menardii* tests were then cleaned in turns through sonication in alternating ethanol and Milli-Q + water, and then oven-dried at 40 °C.

*Cross section preparation.* For some specimens (T_YTT2_, T_YTT3_, T_YTT4_, T_YTT5_), the last chamber was broken with a fine needle blade in ethanol and then each fragment was fixed to a needle by cyanoacrylate glue on its edge. For the T_YYT1_ and T_IND_ specimens, lifting out Ga-Focused Ion Beam (FIB) sections of thin lamellae perpendicularly to the wall surface from the last and penultimate chambers was performed using the FEI STRATA DB 235 FIB system operating at the IEMN (Lille, France), optimizing the standard TEM lamella preparation^48^ for metal XRF analyses, as described below. A 1 µm thick carbon deposit by ion beam was used for shielding our zones of interest. A non-metallic SEM-glue was employed for welding the FIB lamellae both during their lift-out and their fixation to a silicon FIB-TEM grid. At the end, the lamellae were cleaned of re-sputtered damaged material using a low voltage grazing incidence Ga ion beam (5 kV), while a 30 kV one was used for all the other steps of the sample preparation.

### Micro-XRF at the ID21 beamline and respectively nano-XRF at the ID16B beamline of the ESRF

*Setups.* Micro and nano-XRF imaging were performed at ESRF’s ID21^49^ and ID16B^50^ beamlines respectively, which probe the elemental composition and speciation in the tender (2 to 8 keV) and hard (6–30 keV) X-ray regions. These undulator beamlines are commonly equipped with a fixed-exit double mirror system for harmonic rejection, a fixed-exit double crystal monochromator (Kohzu Precision Co., Ltd, Japan) with an Si (111) crystal pair providing a monochromatic X-ray beam with an energy resolution of Δ*E*/*E* ≈ 2 × 10^−4^ and a Kirkpatrick–Baez mirror system for X-ray beam focusing. On ID21 the focused beam size was 0.68 µm × 0.41 µm (8·10^10^ ph/s) at 7.3 keV and 0.75 µm × 0.31 µm (5·10^9^ ph/s) at 2.5 keV (horizontal × vertical). On ID16B, the focused beam size was 55 nm × 60 nm with a flux of 5·10^11^ ph/s at 17.4 keV. On both beamlines, the flux was monitored by a drilled photodiode placed upstream from the sample. Fluorescence was detected by single (ID21) or multi (ID16B) element Silicon Drift Diode Detectors (Bruker XFlash 5100, 80 mm^2^ active area and RaySpec 2 × 3 × 80 mm^2^). On ID16B, the needle was mounted and rotated in order to get (i) circular shapes for the pores of the fragments of T_YTT2_, T_YTT3_ and T_YTT4_ guaranteeing a parallel orientation of the beam to the axis of growth (ii) the thinnest and most continuous image of the transverse sections of T_YTT5_ in the XRF maps, guaranteeing a beam perpendicular to the axis of growth.

The fluorescence spectra were collected in “zap” continuous scanning mode, where the sample was raster-scanned in front of the focused beam and the detectors continuously collected spectra. Consequently, a map of 10 × 10 µm^2^ may produce up to 4·10^4^ spectra, and take anything between a few minutes and up to 10 h. Recorded fluorescence lines of elements of atomic number Z ≥ 12 (Mg) were deconvoluted using the ESRF freely available PyMCA data analysis code (https://sourceforge.net/projects/PyMCA)^51^. The code fits characteristic element fluorescence lines and performs qualitative and quantitative analyses. XRF maps provide raw counts of element lines in each pixel. The raw counts of the profiles perpendicular to the two test surfaces were summed up to produce high statistics spectra for quantitative analysis parametrization.

*Semi-quantitative analyses.* When studying element correlations and the banding effect as a function of the wall structure, the element fluorescent count ratios can be directly used, as the ratios preserve their variations, up to a constant (function of the set-up, excitation energy, and absorption). When quantifying elemental ratios of ultra-thin sections, the *S*_*i*_ detected photon counts are related to the trace element concentrations *c*_*i*_ by the following fundamental parameter equation:

1 $$c_{i} = S_{i} \cdot \frac{{A_{i} }}{{\sigma_{i} \cdot \varepsilon_{i} }}$$

with *A* the atomic mass, σ the fluorescence cross section of element *i* as a function of the incident energy and ε the detector efficiency for the fluorescence lines of element *i*. While normal absorption of the fluorescence lines (K_α_ mainly) of Ca, Zn and Mn through 1 µm-thick FIB samples is less than ca. 4%, and thus reasonably approximated as an ultra-thin section, it reaches values of 24.8%, 33.3% and 13.2% for Ca, Mn and Zn, respectively, through a 10 µm-thick CaCO_3_ slab of 15% porosity (ρ ≈ 2.3 g/cm^3^^52^). Therefore, the semi-quantification of the bulk Mn/Ca and Mn/Zn elemental ratios from a full test thickness of ca. 10 µm implies matrix corrections. In fact, lines emitted from 10 µm thick samples in our specific detection geometry (see ID16B setup above) are both enhanced and absorbed due to the extra sample thickness. The overall effect of these combined corrections is an increase in the raw counts for Ca, Mn, and Zn. The PyMCA fit introduces corrections based on the integrated outcome of these effects, and calculates mass fractions, based on the concentration of the fitted elements versus the matrix composition. Values of the Mn/Ca and Mn/Zn ratios were thus semi-quantitatively evaluated in wt% applying PyMCA’s built-in mass fraction correction. This procedure is valid to better than 4% for Mn/Ca and Mn/Zn ratios for the specific case of 10 µm-thick CaCO_3_ slab of 15% porosity and 17° grazing exit angle. The quantification was based on the average XRF spectra (compiled as in Supplementary Fig. S1 at 17.4 keV) measured for three *Globorotalia menardii* extracted from the YTT (T_YTT2_, T_YTT3_ and T_YTT4_ samples). Bulk Mn/Ca and Mn/Zn elemental ratios of the three different foraminifer values have statistical uncertainties better than 3.2% but a much higher standard deviation so only this value was reported in the main text.

*Statistics.* Following the standard Poisson uncertainties, the statistical error bars of all lines are estimated as the square-root of the raw counts. Actually, the number of counts, S, and its uncertainty*, σ*_*S*_ are output by the PyMCA fitting procedure. The count rate ratio S_X_/S_Y_, has an uncertainty, $\sigma_{{\left( {S_{X} /S_{Y} } \right)}}$, as usual:

2 $$\sigma_{{\left(\frac{{S_{X} }}{{S_{Y} }}\right)}} = \frac{{S_{X} }}{{S_{Y} }} \cdot \sqrt {\left( {\frac{{\sigma_{{S_{X} }} }}{{S_{X} }}} \right)^{2} + \left( {\frac{{\sigma_{{S_{Y} }} }}{{S_{Y} }}} \right)^{2} }$$

so the relative uncertainty of any trace or minor element/Ca ratio is, in first approximation, close to that of the trace or minor element, as the dominating Ca line has very small uncertainties. Error propagation statistical estimates were applied to evaluate the uncertainty for the second derivative of a count rate in a particular position, x, along a profile using its finite difference approximation:

3 $$\left( {\frac{{S_{X} }}{{S_{Y} }}} \right)^{\prime \prime } = \frac{{\left( {\frac{{S_{X} }}{{S_{Y} }}} \right)(x + \Delta x) - 2 \cdot \left( {\frac{{S_{X} }}{{S_{Y} }}} \right)(x) + \left( {\frac{{S_{X} }}{{S_{Y} }}} \right)(x - \Delta x)}}{{\Delta x^{2} }}$$

where ∆x is the width of a step. Doing so, the relative uncertainty ε of the second derivative of the ratio is approximated by:

4 $${{\varepsilon }_{{{\left( \frac{{{S}_{X}}}{{{S}_{Y}}} \right)}^{\prime \prime }}}}=\frac{{{\sigma }_{\left( \frac{{{S}_{X}}}{{{S}_{Y}}} \right)}}}{\left( \frac{{{S}_{X}}}{{{S}_{Y}}} \right)}=2\cdot {{\varepsilon }_{_{\left( \frac{{{S}_{X}}}{{{S}_{Y}}} \right)}}}$$

which is twice the value of the relative elemental ratio uncertainty.

### Supplementary information

Supplementary information.

## Appendix 1

### The intra-test structure: ICL-POZ-OCL-GC inherited from the morphogenesis of the test of *G. menardii*

*G. menardii*, as with the majority of planktic foraminifers, undergoes calcitic test construction via the sequential addition of chambers (Fig. 2). Chamber formation starts with the extrusion from the aperture of a cytoplasmic bulge (CB) delimited by a protective cytoplasmic envelope (CE), sketching the shape of a new chamber^2^. The calcareous wall is likely generated through a specific calcification sequence that is supposed to be implemented in an extracellular privileged space (the calcification site) located between the CB and CE and supersaturated with respect to CaCO_3_^4,34^.

In the seminal work carried out by Hemleben et al.^28^ on the test morphogenesis of *G. menardii*, it was observed by scanning electron microscopy (SEM) that the early calcification stage of a new chamber’s wall starts within a single soft layer that may only be about 1.5 µm thick. This organic material, loosely packing individual crystallites, was called a precursor layer and was assimilated to the organic template previously called “Primary Organic Membrane (POM)” (nowadays called Primary Organic Sheet, POS)^34^. At higher resolution, Transmission Electron Microscope images of the POS display it as a spongy layer composed of a complex branched organic network ~ 700 nm thick^11,28^. Since the POS is not a single laminar organic structure, but rather a dispersed organic framework, in this work we prefer to use the term Primary Organic Zone (POZ) instead of POS.

The subsequent stage of wall growth is a “bipolar” (bi-directional) precipitation of two calcite layers (bilayer structure), on either side of the precursor layer. The inner calcitic layer (ICL) and the outer calcitic layer (OCL) consist of small (submicron) calcite crystallites^28^ (Fig. 2C). The intra-test inner calcitic layer (ICL), POZ and outer calcitic layer (OCL) microarchitecture of the wall, the “ICL-POZ-OCL”, is inherited from the wall build-up of a new chamber and constitutes the incipient bilayer wall, clearly visible by SEM (Fig. 2C).

In the older chamber, the outer multi-layer structure of the wall reflects the sequential addition of chambers during the ontogeny, with a new OCL covering the whole test structure each time a new chamber is added^28^ (Fig. 2). A final calcitic layer, composed by larger well-developed euhedral calcite, might be deposited upon the chambers of the last whorl of the test at the end of the test growth, once the last chamber is formed and prior to gamete release^2,28^. This is referred to as the gametogenic crust (GC), which is a specific feature of planktic foraminifers. Laboratory experiments with non-spinose foraminifera suggested that crust addition might be a function of temperature reduction as foraminifera settle through the water column^2^. However, more recently, crust addition was observed in *Neogloboquadrina dutertrei* and *N. incompta* maintained in culture at constant temperature, showing that the mechanisms responsible for GC formation remain to be resolved^8^.
