# Supplementary material for: Nanoscale trace metal imprinting of biocalcification of planktic foraminifers by Toba’s super-eruption
Source: Sci Rep. 2020 Jul 3;10:10974. doi: 10.1038/s41598-020-67481-w (PMC7335162; doi:10.1038/s41598-020-67481-w)
Supplement: Supplementary file 1 — Supplementary information. [file 41598_2020_67481_MOESM1_ESM.pdf]

SUPPLEMENTARY MATERIALS

Nanoscale trace metal imprinting of biocalcification of planktic foraminifers by Toba's super-eruption

Selective metal incorporation in foraminifer's tests

L. Lemelle[1], A. Bartolini[2*], A. Simionovici[3†], R. Tucoulou[4], W. De Nolf[4], F. Bassinot[5], T. de Garidel-Thoron[6]

[1]Univ Lyon, ENS de Lyon, Univ Claude Bernard, CNRS, LGL-TPE, 46 allée d'Italie, F-69342 Lyon, France, Laurence.lemelle@ens-lyon.fr

[2]Muséum national d'Histoire Naturelle, Département Origines & Evolution, CR2P MNHN, CNRS, Sorbonne Université, 8 rue Buffon CP38 75005 Paris, France, bartolini@mnhn.fr

[3]ISTerre, Univ. Grenoble Alpes, CNRS, CS 40700, 38058 Grenoble Cedex 9, France,

[†]Institut Universitaire de France (IUF), alexandre.simionovici@univ-grenoble-alpes.fr

[4]ESRF-The European Synchrotron Research Facility, ID21 / ID16B beamlines, 71 avenue des Martyrs, CS40220, 38043 Grenoble Cedex 9, France, tucoulou@esrf.fr, wout.denolf@esrf.fr

[5]Institut Pierre-Simon Laplace/Laboratoire des Sciences du Climat et de l'Environnement, UMR 8212, CEA-CNRS-UVSQ, 91190 Gif-sur-Yvette, France, franck.bassinot@lsce.ipsl.fr

[6]Aix-Marseille Univ., CNRS, IRD, Collège de France, INRAE, CEREGE, 13545 Aix-en-Provence cedex 4, garidel@cerege.fr

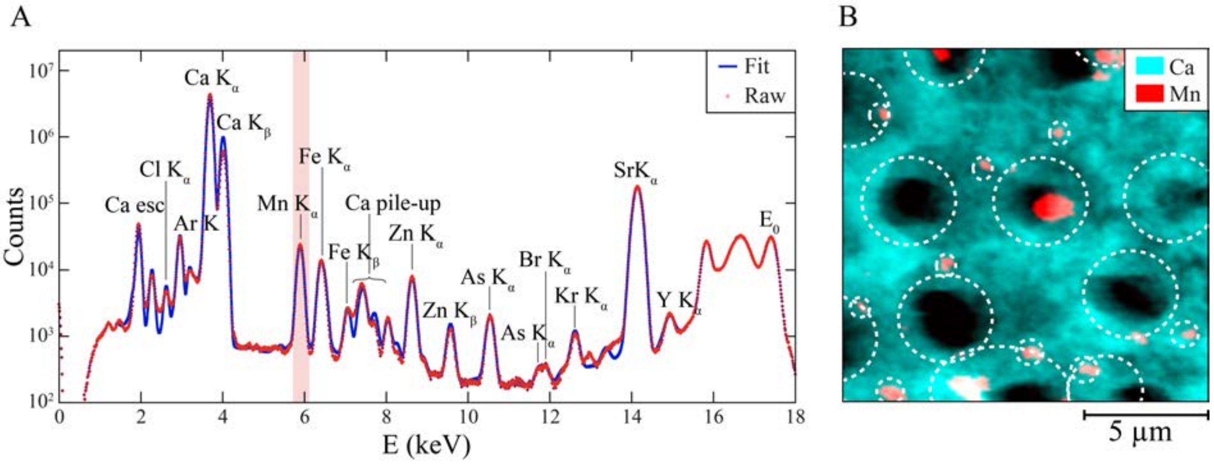

Fig. S1. **Integrated nano-XRF analysis of the whole wall.** (A) Average XRF spectrum (red dots) compiled from a nano-XRF map recorded at 17.4 keV (dwell-time is 0.5 s, scan step size is 100 nm) on a fragment of the last chamber wall of a *Globorotalia menardii* extracted from the YTT of the specimen T_{YTT2} (BAR94-25, 317 cm depth). The main K_α lines of elements are reported with the Mn K_α line in the red zone. The fit (blue line) and the K_α raw counts were compiled using PyMCA. (B) The Ca K_α line map (in blue) superimposed on that of Mn K_α line map (in red) is displayed. Scale bar is 5 µm. Only the homogeneous part of the wall was selected, the pores and surface impurities (delineated by dashed white lines) were excluded from the average.

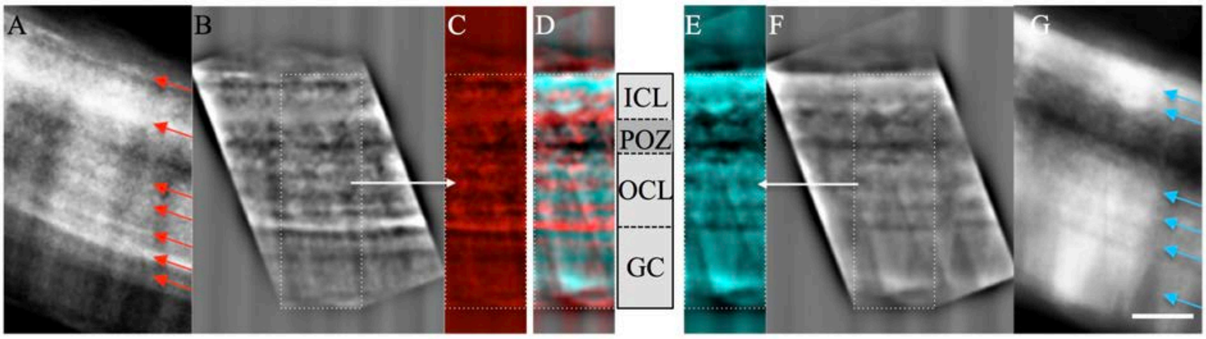

Fig. S2. Mn and Ca nano-banding imprinted on the wall structure. (A and G) High spatial resolution map of the Mn and Ca K_α lines shown in Fig. 3. Scale bar is 4 µm. (B and F) The positions of the brightest bands are indicated by plain lines. Same image after rotation and Fiji-filtering (CLAHE and FFT-bandpass filtering of large structures down to 25 pixels and small structures up to 3 pixels) to enhance the local contrasts of the nanobanding in (A and G). (C and E) Focus on the same rectangular zone (white dashed array an B and F) color-coded in red for Mn and turquoise for Ca that are merged in (D) and displays Mn nano-banding in opposite phase to the Ca nano-banding except for the POZ. The ICL-POZ-OCL-GC wall growth structure is indicated as gray boxes.

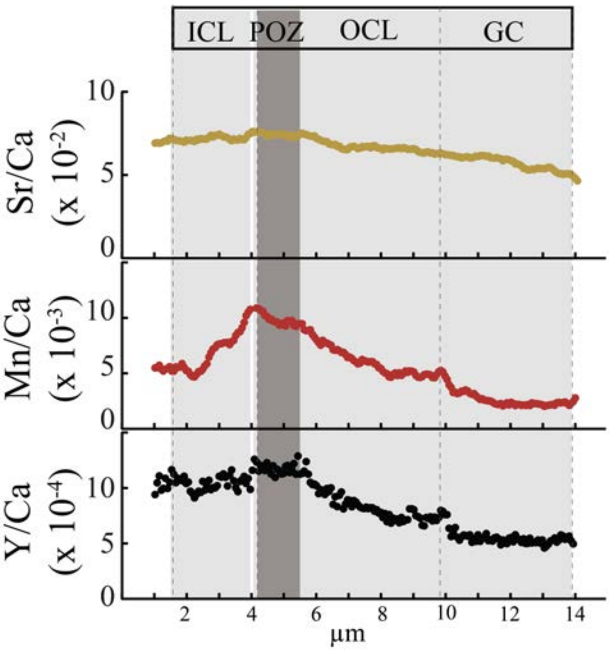

Fig. S3. **Correlation between the Sr/Ca, Mn/Ca and Y/Ca profiles.** The profiles have been measured on the *G. menardii* last chamber T_{YTT5} fragment oriented parallel to the axis of the ICL-POZ-OCL-GC wall growth structure (see corresponding SE-SEM image in Fig. 2C), and averaged along a 21 pixel-wide rectangle displayed by a white dashed line box in the maps of Ca and Mn K_α lines recorded at 17.4 keV on ID 16B ESRF (see Fig. 3). The ICL-POZ-OCL-GC structure is indicated as gray boxes. Note that the POZ yields the highest values of Sr/Ca, Mn/Ca and Y/Ca, while the GC the lowest ones. The Mn/Ca and Y/Ca profiles are quite similar.

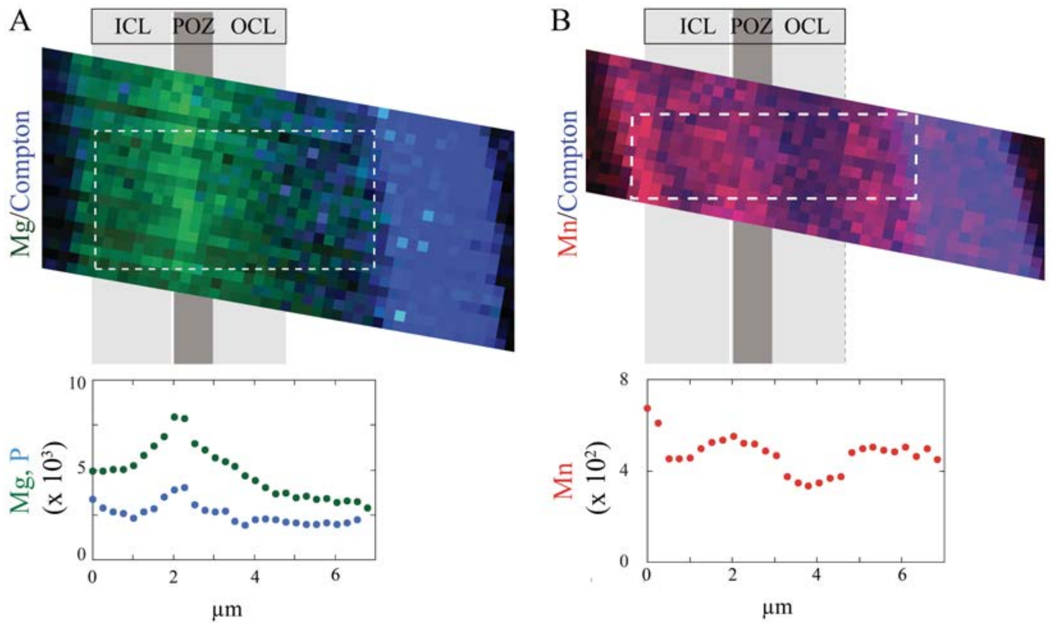

Fig. S4. **Mg-P-Mn distributions imprinted on the wall growth structure from the last chamber of a modern *Globorotalia menardii* specimen** T_{IND} **(Gyrafor-B, St.C, T3N4-2F) seen by micro-XRF**. The wall ICL-POZ-OCL structure is indicated by gray boxes (see the corresponding SEM image of the T_{IND} FIB section in Fig. S5). (A) High spatial resolution map of the Mg K_α line recorded at E_{inc}=2.47 keV on ID 21 ESRF (10 s dwell-time, 250 nm step size, 300×300 nm^2 beamsize) and the Mg and P profiles measured on the T_{IND} FIB section (Fig. S5). Spectra were averaged along a 16 pixel-wide rectangle. Data is reported in counts of fluorescence. (B) High spatial resolution map of the Mn K_α line measured at E_{inc}=7.3 keV on the same sample and the corresponding Mn profile set-up (5 s dwell-time) was averaged along an 8 pixel-wide rectangle. Structures are broadened at E_{inc}=7.3 keV due to the larger beamsize (approx. 1.2 μm). The Compton map was superimposed to delimit the contour of the wall (gray boxes), as the thick low Z carbon deposit on the external surface (used for shielding the zone of interest during the FIB ablation) is imaged as a bright layer. Error bars are in the line widths.

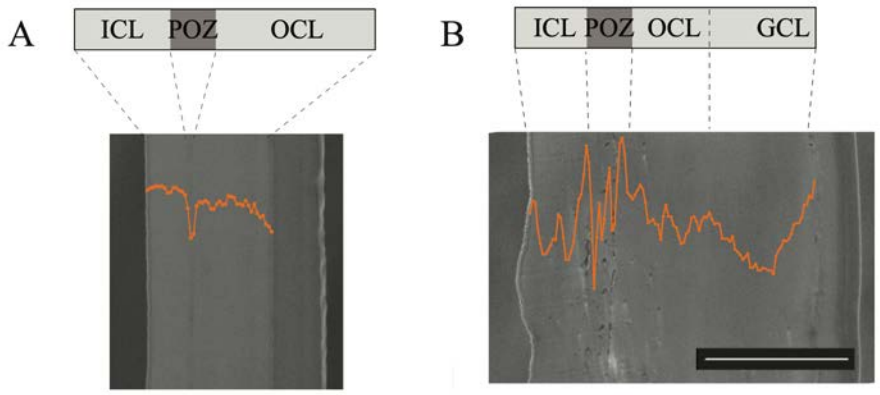

Fig. S5. **Comparison of the wall structure of the last chamber from the modern specimen of *Globorotalia menardii* T_{IND} and the specimen coming from the YTT level, T_{YTT1}**. SE-SEM of FIB sections, cut perpendicularly to the last chamber's wall surface. Orange dots show the gray levels measured along a 1 µm - wide line perpendicular to the wall's surface. Scale bar is 5 µm. (A) Image from a modern specimen collected by nets in Indian Ocean subsurface seawater (Gyrafor-B, St.C, T3N4-2F). The thick dark gray protective carbon strip (used for shielding the zone of interest during the FIB ablation, see Materials and Method) is well visible next to the OCL. The ICL-POZ-OCL wall structure is indicated as gray boxes. Note the absence of the GC, implying that this individual had not fully completed its ontogenic development. (B) Image from the specimen T_{YTT1} picked from the YTT level (BAR94-25 core, 307 cm depth). The ICL-POZ-OCL-GC structure is indicated as gray boxes. The protective dark gray carbon strip is clearly visible next to the GC.

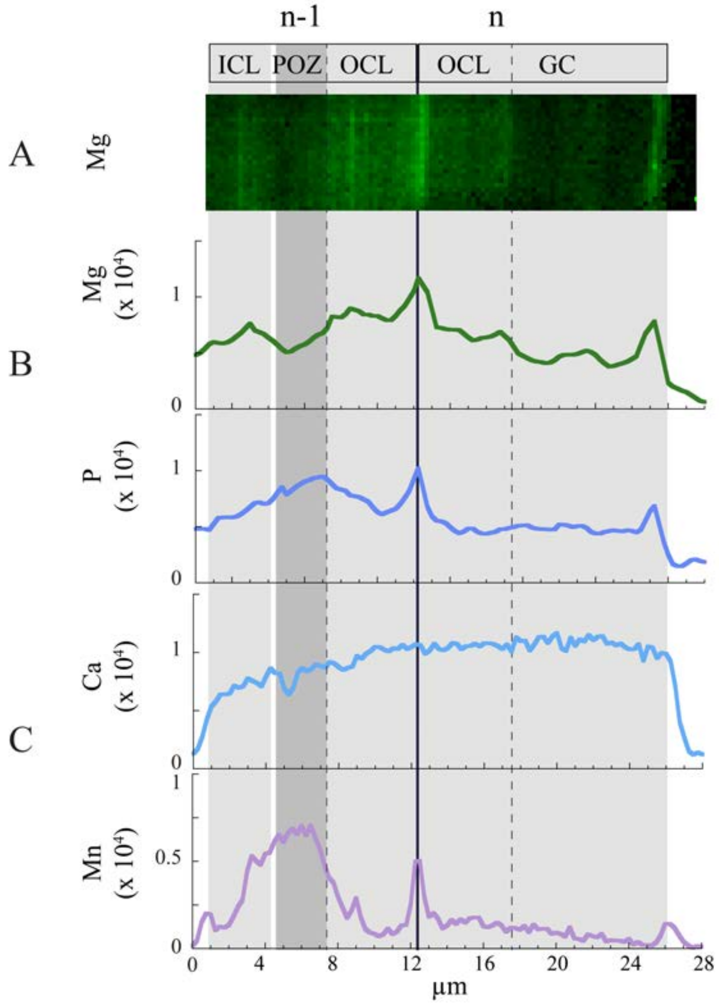

Fig. S6. Ca-Mn-P-Mg distributions imprinted on the calcitic structure of the wall from the last but one (n-1) chamber of the *Globorotalia menardii* specimen T_{YTT1} (BAR94-25 core, 307 cm depth) seen by micro-XRF. The wall ICL-POZ-OCL-OCL-GC structure is indicated by gray boxes. The wall (ICL-POZ-OCL structure), biomineralized during the formation of the n-1 chamber, is overlayed by a further OCL and the GC biomineralized during and after the formation of the last (n) chamber. The black line in-between the OCLs represents the organic lining. (A) High spatial resolution map of the Mg K_α line recorded at E_{inc}=2.47 keV on ID 21 ESRF (10 s dwell-time, 250 nm step size, 300×300 nm^2 beamsize). (B) The Mg and P profiles measured perpendicularly to the wall surface of the FIB section. (C) The Mn and Ca profiles measured at E_{inc}=7.3 keV on the same sample and set-up (5 s dwell-time) were averaged along an 8 pixel-wide rectangle. Structures are broadened at E_{inc}=7.3 keV due to the larger beamsize (approx. 1.2 µm). Error bars are in the line width.
